# Utility Metrics for Evaluating Synthetic Health Data Generation Methods: Validation Study

**DOI:** 10.2196/35734

**Published:** 2022-04-07

**Authors:** Khaled El Emam, Lucy Mosquera, Xi Fang, Alaa El-Hussuna

**Affiliations:** 1 School of Epidemiology and Public Health University of Ottawa Ottawa, ON Canada; 2 Children's Hospital of Eastern Ontario Research Institute Ottawa, ON Canada; 3 Replica Analytics Ltd Ottawa, ON Canada; 4 Open Source Research Collaboration Aarlberg Denmark

**Keywords:** synthetic data, data utility, data privacy, generative models, utility metric, synthetic data generation, logistic regression, model validation, medical informatics, binary prediction model, prediction model

## Abstract

**Background:**

A regular task by developers and users of synthetic data generation (SDG) methods is to evaluate and compare the utility of these methods. Multiple utility metrics have been proposed and used to evaluate synthetic data. However, they have not been validated in general or for comparing SDG methods.

**Objective:**

This study evaluates the ability of common utility metrics to rank SDG methods according to performance on a specific analytic workload. The workload of interest is the use of synthetic data for logistic regression prediction models, which is a very frequent workload in health research.

**Methods:**

We evaluated 6 utility metrics on 30 different health data sets and 3 different SDG methods (a Bayesian network, a Generative Adversarial Network, and sequential tree synthesis). These metrics were computed by averaging across 20 synthetic data sets from the same generative model. The metrics were then tested on their ability to rank the SDG methods based on prediction performance. Prediction performance was defined as the difference between each of the area under the receiver operating characteristic curve and area under the precision-recall curve values on synthetic data logistic regression prediction models versus real data models.

**Results:**

The utility metric best able to rank SDG methods was the multivariate Hellinger distance based on a Gaussian copula representation of real and synthetic joint distributions.

**Conclusions:**

This study has validated a generative model utility metric, the multivariate Hellinger distance, which can be used to reliably rank competing SDG methods on the same data set. The Hellinger distance metric can be used to evaluate and compare alternate SDG methods.

## Introduction

Interest in synthetic data generation (SDG) has recently grown. Synthetic data are deemed to have low privacy risks in practice because there is no one-to-one mapping between synthetic records and real people [1-8]. Recent evidence supports the low privacy risk claim [9]. This enables synthetic data to be used and shared for secondary purposes without the need for further consent [10]. In addition to meeting privacy requirements, synthetic data must also have sufficient utility. This utility can be evaluated using utility metrics. Utility metrics are important in hyperparameter tuning of the generative models during training and communicating data quality to the synthetic data users and for researchers and analysts when ranking different SDG methods to select the best one. Our focus in this paper is on the ranking of SDG methods.

Utility metrics can be defined as narrow or broad [11]. Narrow metrics are specific to an analysis that is performed with the synthetic data and are also sometimes referred to as workload-aware utility metrics. For example, if the objective is to build a model between a predictor and a binary outcome, controlling for multiple confounders, then the difference in accuracy of real versus synthetic model predictions on holdout data sets would be a workload-aware utility metric. There have been multiple studies evaluating narrow metrics [12-16]. Narrow metrics represent what the data user is ultimately interested in. Data users want synthetic data sets that score highly on narrow utility metrics.

Researchers and analysts need to rank SDG methods. For example, a developer of an SDG method may use an ensemble of techniques and then select the one with the highest utility as the final result, or analysts may evaluate multiple SDG methods available in the marketplace to select one for their own projects. However, all workloads are typically not known in advance. Therefore, researchers and analysts cannot evaluate the narrow utility of the SDG methods directly. Instead, they need to use broad utility metrics during the SDG construction and evaluation process. A key requirement is that broad utility metrics are predictive of narrow utility metrics for plausible analytic workloads.

Some studies utilized broad metrics, for example, to compare and improve SDG methods [17-19]. However, many of the broad utility metrics currently used have not been validated. This means that there is a dearth of evidence demonstrating that they are predictive of narrow utility metrics under realistic decision-making scenarios.

The realistic decision-making scenario that we are considering here is the comparison and ranking of SDG methods. Finding the best SDG method is becoming a more common need in the literature, and we need reliable metrics to be able to draw valid conclusions from these comparisons. Furthermore, in practice, users of SDG methods need to have good metrics to select among a number of these methods that may be available to them.

Utility metrics can be classified in a different way, which is relevant for our purposes. They can pertain to a specific synthetic data set or to the generative model (“data set–specific” and “model-specific” utility metrics). Because SDG is stochastic, the utility of synthetic data sets generated from the same generative model will vary each time the generative model is run, and sometimes that variation can be substantial. Data set–specific utility metrics are useful when one wants to communicate how good the particular generated data set is to a data user. However, these utility metrics are not necessarily useful, for example, for comparing different generative models because of the stochasticity. A model-specific utility metric reflects the utility of the generated synthetic data sets on average, across many data sets that are generated from the same model. Such a metric is more useful in our context, where we want to compare and rank SDG methods.

Our focus in the current study is to perform a validation study of broad model-specific utility metrics for structured (tabular) health data sets. While there have been evaluations of generative model utility metrics in the past, these have focused on images rather than structured data [20]. One previous more relevant evaluation considered propensity mean squared error (pMSE) [21,22] as a model utility metric whereby its correlation with binary prediction accuracy on synthetic data was empirically assessed [23]. The authors found that when used as a broad model-specific utility metric, by averaging across multiple synthetic data sets, this metric had a moderate correlation with narrow model-specific utility metrics. However, the correlation between a broad metric and a narrow metric across many data sets for a single SDG technique does not reflect an actual decision-making scenario. In practice, we have a single data set and multiple SDG techniques. Therefore, the extent to which the results from that previous study would be informative to our scenario of interest is unclear.

We build on this previous work by considering other types of broad model-specific utility metrics beyond pMSE and adjust the methodology to more closely model a practical decision-making scenario of an analyst selecting among multiple SDG methods to identify the one with higher narrow utility on logistic regression prediction tasks. This type of prediction task is used often in health research.

## Methods

The protocol for this study was approved by the CHEO Research Institute Research Ethics Board (number CHEOREB# 21/144X). Our objective was to answer the following question: Which broad model-specific utility metrics can be used to rank SDG methods in terms of the similarity of prediction performance between real and synthetic data? In the following sections we describe the methods that were followed.

### Data Sets

For our analysis, we used the 30 health data sets that are summarized in Appendix S1 in Multimedia Appendix 1. These data sets are available publicly or can be requested from the data custodians. Many of these data sets have been used in previous evaluations of SDG techniques [12,15,23], and therefore we can ensure some consistency across studies in this domain. These data sets also represent a heterogeneous set of clinical situations (providing care, observational studies, clinical trials, and registries), a wide range of data set sizes (87-44,842 patients), and variation in data set complexity (as measured using average variable entropy), which allow our evaluations to be more generalizable.

### The Broad Utility Metrics Considered

Broad utility metrics compare the joint distributions of the real and synthetic data sets. Many metrics have been proposed to compare joint distributions [24]. We only focus on 6 multivariate metrics that have been used in previous work to evaluate the utility of synthetic data sets.

### Maximum Mean Discrepancy

The maximum mean discrepancy metric is one way to test whether samples are from different distributions [25]. In our implementation, we used a radial basis function kernel. This metric has been applied to assess the utility of synthetic health data [26,27]. It is also widely used in the training of deep learning models and evaluation of the quality of synthetic data. Recent work on a recurrent Generative Adversarial Network (GAN) and recurrent conditional GAN made use of maximum mean discrepancy to assess whether the time series generated by the generative model implicitly learns the distribution of the true data [28]. Another study evaluated synthetic data in the smart grid context, in which a GAN is used to learn the conditional probability distribution of the significant features in the real data set and generates synthetic data based on the learnt distribution [29].

### Multivariate Hellinger Distance

The Hellinger distance [30] has been shown to behave in a consistent manner as other distribution comparison metrics, specifically in the context of evaluating disclosure control methods [31], when comparing original and transformed data.

The Hellinger distance can be derived from the multivariate normal Bhattacharyya distance and has the advantage that it is bound between 0 and 1 and hence is more interpretable [32]. We constructed Gaussian copulas from the original and synthetic data sets [33] and then computed the distance between them. The concept of comparing the distance between 2 multivariate Gaussian distributions has been used to train GAN-based SDG methods [34]. Additional details on its calculation are provided in Appendix S2 in Multimedia Appendix 1.

### Wasserstein Distance

The *W*_1_ Wasserstein distance [35] is often applied to the training of GANs [36]. It has resulted in a learning process that is more robust by alleviating the vanishing gradient issue and mode collapse.

While GANs have been used extensively as an SDG technique, they very often still have trouble capturing the temporal dependency of the joint probability distributions caused by time-series data. The conditional sig-Wasserstein GANs proposed for time series generation is aimed at addressing this problem [37]. Here, the authors combine the signature of paths, which statistically describe the stream of data, and the *W*_1_ distance, to capture the joint law of time series. By employing the sig-W as the discriminator, sig-Wasserstein GAN shows an ability to generate realistic multidimensional time series. Additional details on its calculation are provided in Appendix S2 in Multimedia Appendix 1.

### Cluster Analysis Measure

The original cluster metric [21] was first purposed as a global measure of the data utility of original data and masked data. The cluster analysis has 2 steps: first, merge the original data (O) and masked data (M); then, given a certain number of groups G, perform cluster analysis on the merged data. The measure can be calculated as:

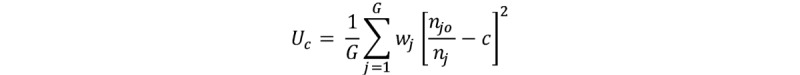



Where, *n_j_* denotes the number of observations in the jth cluster and *n_jo_* denotes the number of observations in the jth cluster that are from the original data (O). The c value is defined as:

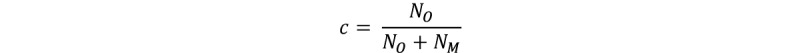



A large *U_c_* value indicates the disparities of the underlying latent structure of the original and masked data. The weight *w_j_* can reflect the importance of certain clusters. This cluster analysis measure is used in the evaluation of synthetic data by simply replacing the original data with real data and the masked data with synthetic data [17].

### Distinguishability Metrics

These broad metrics are based on the idea of training a binary classifier that can discriminate between a real and synthetic record [38,39]. That ability to discriminate is converted into a score.

A propensity mean square error metric has been proposed to evaluate the similarity of real and synthetic data sets [21,22], a perspective adopted from the propensity score matching literature [40], which we will refer to as *propensityMSE*. To calculate the *propensityMSE*, a classifier is trained on a stacked data set consisting of real observations labelled 1 and synthetic observations labelled 0. The *propensityMSE* score is computed as the mean squared difference of the estimated probability from the average prediction where it is not possible to distinguish between the 2 data sets. If the data sets are of the same size, which is the assumption we make here, and indistinguishable, then the average estimate will be 0.5.

Another related approach that has been used to evaluate the utility of synthetic data is to take a prediction perspective rather than a propensity perspective. This has been applied with “human discriminators” by asking a domain expert to manually classify sample records as real or synthetic [41-43]. This means that a sample of real records and a sample of synthetic records are drawn, and the 2 sets are shuffled together. Then the shuffled records are presented to clinicians who are experts in the domain, and they are asked to subjectively discriminate between the records by indicating which is real versus synthetic. High distinguishability only occurs when the human discriminator can correctly classify real and synthetic records.

The use of human discriminators is not scalable and therefore we can use machine learning algorithms trained on a training data set and that make predictions on a holdout test data set. This approach mimics the subjective evaluations described above. We will refer to this metric as *predictionMSE*. Also note that this calculation is different from the calculation of *propensityMSE* where the training data set is also used to compute the probabilities. Additional details on the calculations are provided in Appendix S2 in Multimedia Appendix 1.

### Workload Aware (Narrow) Metrics

To assess whether the utility metrics are useful, we evaluated whether they can accurately rank SDG methods on workload aware metrics. This section describes these workload aware metrics.

We built a logistic regression (LR) model for each data set. LR is common in health research, and a recent systematic review has shown that its performance is comparable to that of machine learning models for clinical prediction workloads [44]. Furthermore, an evaluation of the relative accuracy of LR models compared to that of other machine learning techniques, such as random forests and support vector machines, on synthetic versus real data sets across multiple types of SDG methods showed that LR models are only very slightly different [23]. Therefore, we would expect that the results using LR would provide broadly applicable and meaningful results.

We evaluated the prediction accuracy using 3-fold crossvalidation. Accuracy was measured using the area under the receiver operating characteristic curve (AUROC) [45] and the area under the precision-recall curve (AUPRC) [46]. For outcomes that had multiple categories, we used the average of pairwise AUROC values [47]. The AUPRC values for multicategory outcomes were macroaveraged. This was performed for each real and each synthetic data set.

To assess the similarity between the AUROC and AUPRC for the real and synthetic data sets, we computed the absolute difference between them. This provides a measure of how similar the real results are to the synthetic results.

### Evaluation Methodology

For each of the 30 real data sets, we generated 20 synthetic data sets. The utility metrics and the absolute AUROC difference and absolute AUPRC difference were computed on each of the 20 synthetic data sets, and each of these was averaged. Therefore, for each of the data sets, we had 1 average utility metric value for each of the 6 utility metrics, 1 average AUROC difference value, and 1 average AUPRC difference value. These values are tabulated in Appendix S3 and S4 in Multimedia Appendix 1.

### SDG Methods

The main hypothesis that we wanted to test was whether the utility metrics can be used to rank the SDG methods by their AUROC and AUPRC differences. The SDG methods were chosen to achieve representativeness, applicability, and variation.

1. Representativeness. The methods should reflect those that are often used in the community of practice and by researchers.
2. Applicability. The methods are those that an analyst would likely want to compare and select from to be consistent with our motivating use case.
3. Variation. The utility results among the chosen SDG methods should have variation sufficient for utility metrics to detect differences.

Three generative models were used: conditional GAN [48], a Bayesian network [49], and a sequential synthesis approach using decision trees [19]. The Bayesian network implementation uses a differential privacy approach. These 3 methods were selected for the following reasons: they each represent a class of methods that is often used in the literature (eg, sequential synthesis has been used on health and social sciences data [50-58], as well as Bayesian networks [26,59] and GANs [2,60,61]), they use very different approaches and therefore represent plausible SDG methods that an analyst would want to compare, and they are expected to exhibit large utility level variation given that different SDG methods tend to be better at modeling certain types of variables and relationships. For these 3 reasons, this set of SDG methods was suitable for this study on validating utility metrics.

### Individual Utility Metric Ranking

We used the Page test to determine whether the utility metric prediction was correct [62]. For that, we specified 3 groups for each utility metric: an “L” group where the utility metric indicates low utility (ie, has the highest value since they are all distance-type metrics), an “H” group where the utility metric indicates high utility (ie, has the lowest value), and an “M” group in the middle. This process is repeated for each utility metric. For any particular data set, the generative model with the lowest utility is put in the “L” group, the generative model with the highest utility is put in the “H” group, and the third generative model is in the “M” group. Each generative model in a group is replaced with its AUROC or AUPRC difference value, depending on which workload aware metric is under evaluation.

The null hypotheses we were testing are therefore that:

H0_AUROC_: median(AUROC_Diff_L_) = median(AUROC_Diff_M_) = median(AUROC_Diff_H_)

H0_AUPRC_: median(AUPRC_Diff_L_) = median(AUPRC_Diff_M_) = median(AUPRC_Diff_H_)

where the subscript indicates the group. Against the alternatives:

H1_AUROC_: median(AUROC_Diff_L_) ≥ median(AUROC_Diff_M_) ≥ median(AUROC_Diff_H_)

H1_AUPRC_: median(AUPRC_Diff_L_) ≥ median(AUPRC_Diff_M_) ≥ median(AUPRC_Diff_H_)

Where at least one of the inequalities is strict. To compute the test statistic, *L,* the data are put in a matrix with 30 rows, one for each data set, and 3 columns, one for each group. The accuracy scores are used to assign a rank to the values in each row. Then the ranks are summed per column *R_j_* where *j*=1…3. The *L* statistic is then the sum: *L* = *R_1_* + *2R_2_* + *3R_3_*. The larger that value, the greater the evidence supporting the ranking conclusion.

Because of the relatively small sample size, we used an exact test of statistical significance. This also does not make distributional assumptions on the data, and for the number of data sets we have, this gives us a high-powered test.

If the test is significant, then the broad utility metric can be used to correctly rank SDG techniques based on their workload (narrow) metrics. Since we were comparing multiple utility metrics, a Bonferroni adjustment was made to the α level of .05 to account for multiple testing.

The maximum *L* value can be used to identify the utility metric that is best at ranking the SDG methods by prediction accuracy difference. This is particularly useful if more than one metric is found to be statistically significant.

### Aggregate Ranking

Because each utility metric is expected to rank the SDG methods differently, we wanted to test whether an aggregate ranking would provide a better result than any of the individual utility metric rankings. We hoped to find an “ideal” ranking that has minimal distance to each of the individual rankings on the utility metrics. This can be performed for each data set separately, and then the ideal rankings across all the data sets would be evaluated on the Page test. The result would give us the performance of the aggregate ranking, and we can contrast that with the quality of individual utility metric rankings.

The distance we used is the Spearman footrule [63]. With this approach, if method A has a higher ranking than method B more often than not, method A should rank higher than method B in the ideal ranking. Given the relatively small data set, full enumeration rather than an optimization algorithm was used to find the ideal ranking.

Given that the *prediction*MSE and *propensity*MSE are strongly related, the former was removed so as to not give that particular ranking a higher weighting in the aggregation.

## Results

The results of the ranking of the SDG methods are shown in Table 1. All metrics are statistically significant in that the null hypothesis of no difference was rejected. The broad utility metric rankings were close enough to the correct rank, so the relationship was quite strong.

The test statistic, the *L* value, indicates the strength of the ordering of data. The Hellinger distance had the highest *L* value among all the utility metrics, suggesting that it has an advantage in ordering the SDG methods based on their narrow utility metrics.

**Table 1:** Page test results for each of the utility metrics and prediction accuracy

Utility metric	AUROC^a^ difference		AUPRC^b^ difference	
	*L* value	*P* value	*L* value	*P* value
Maximum mean discrepancy	384	.00104^c^	392	<.001^c^
Hellinger distance^d^	398	<.001^c^	409	<.001^c^
Wasserstein distance	392	<.001^c^	403	<.001^c^
Cluster analysis	396,	<.001^c^	405	<.001^c^
Propensity mean squared error	390	<.001^c^	394	<.001^c^
Prediction mean squared error	396	<.001^c^	397	<.001^c^
Aggregate^d^	400	<.001^c^	408	<.001^c^

^a^AUROC: area under the receiver operating characteristic curve.

^b^AUPRC: area under the precision-recall curve.

^c^Statistically significant at a Bonferroni adjusted α level of .05.

^d^Highest metric on the test statistic.

**Figure 1:**
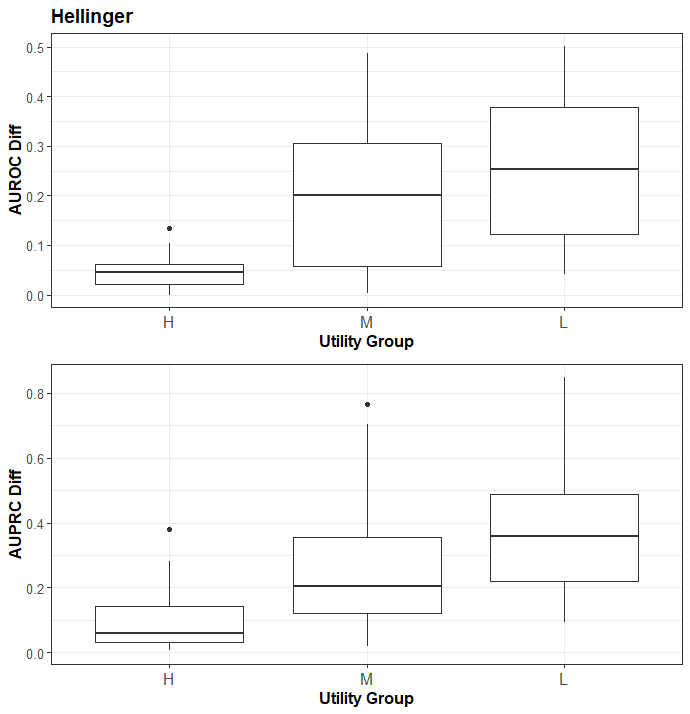
The relationship between the Hellinger distance versus the AUROC and AUPRC. The 3 SDG methods were ordered based on their relative Hellinger distance values into the “H,” “M,” and “L” groups. AUROC: area under the receiver operating characteristic curve; AUPRC: area under the precision-recall curve; SDG: synthetic data generation.

The boxplots in Figure 1 descriptively show the trend for the Hellinger distance. There is a clear trend of higher utility on the narrow AUROC and AUPRC metrics as the Hellinger distances get smaller. The boxplots for the remainder of the utility metrics are included in Appendix S5 in Multimedia Appendix 1, and they all show trends similar to those seen in Figure 1.

**Figure 2:**
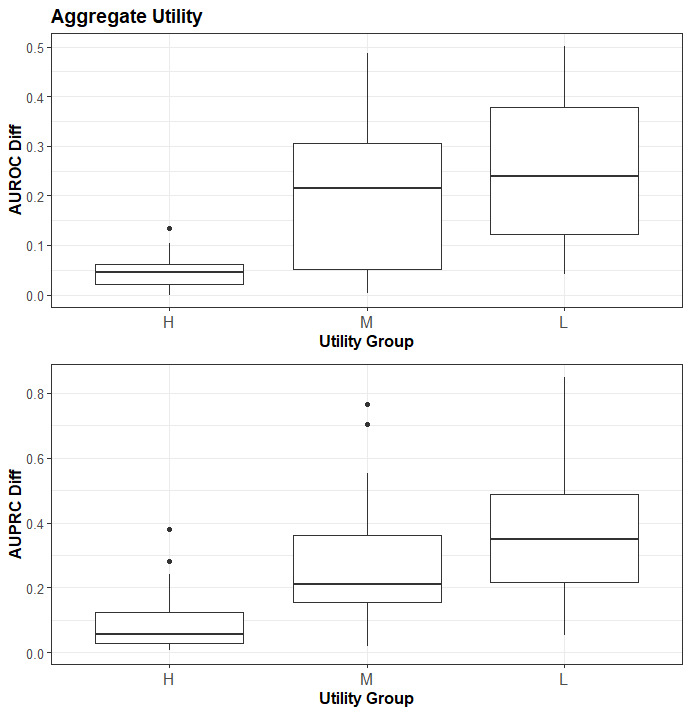
The relationship between the aggregate ranking versus the AUROC and AUPRC. AUROC: area under the receiver operating characteristic curve; AUPRC: area under the precision-recall curve.

The results for the aggregate ranking are shown in Table 1 and Figure 2. As can be seen from the *L* statistic and the boxplots, there is only a slight difference between using the Hellinger distance and the aggregate ranking from 5 different utility metrics. In a post-hoc analysis, we removed each of the metrics in turn in a leave-one-out fashion and recomputed the aggregate rank, but these did not produce better results than the one presented here.

## Discussion

### Summary

The purpose of our study was to identify the most useful, broad generative model utility metrics. These are different from utility metrics calculated for a particular synthetic data set. Generative model utility characterizes the average utility across synthetic data sets that are produced from a generative model. Given the stochasticity of SDG, such utility metrics are more appropriate for evaluating, comparing, and selecting among SDG models on the same real data set. Single synthetic data set utility metrics, on the other hand, are useful for communicating synthetic data utility to a data user because these pertain to the particular synthetic data set that is being shared.

We performed our analysis using 3 types of generative models: a conditional GAN, a Bayesian network, and sequential decision trees. These 3 cover a broad cross-section of types of techniques that are used in practice, which would enhance the applicability and generalizability of the results.

In this study, we evaluated 6 different model-specific utility metrics to determine whether they can be used to rank SDG methods. This is a practical use case that reflects a decision that an analyst using SDG methods would want to make. For example, there are multiple SDG techniques that have been published in the literature, and our ranking results can help an analyst determine the one that would work best on their real data sets.

We defined workload-aware utility as the ability to develop binary or multicategory prediction models that have similar prediction accuracy, measured by the AUROC and the AUPRC, between the real and synthetic data sets. The construction of binary or multicategory prediction models is an often-used analytical workload for health data sets. We used logistic regression to compute the absolute AUROC and AUPRC differences on real and synthetic data sets.

Our results based on an evaluation on 30 heterogeneous health data sets indicated that all the utility metrics proposed in the literature will work well. However, the multivariate Hellinger distance computed over the Gaussian copula has a slight advantage in that it provides better utility ordering. Further examination of an aggregate ranking using multiple utility metrics showed only a negligible difference from the results of the Hellinger distance for the AUROC metric, and therefore the simplicity of a single utility metric would be preferred.

Our results would allow a researcher or analyst to select the SDG method with the highest utility defined in a narrow sense. However, maximum utility does not imply that the privacy risks are acceptably low. As there is a trade-off between utility and privacy, higher utility will increase the privacy risks as well. Therefore, when evaluating SDG methods, it is important to also consider the privacy risks.

Now that we have validation evidence for a broad utility metric, it can be combined with a privacy metric to provide an overall ranking of SDG methods. For example, membership disclosure metrics for generative models [64,65] can be considered along with the multivariate Hellinger distance when SDG methods are ranked. Metrics combining these 2 risk and utility metrics would be a good avenue for future research.

### Limitations

An analyst may need to make other kinds of decisions, such as evaluating different SDG models for the purpose of hyperparameter tuning. Our study did not evaluate that specific use case, and therefore we cannot make broader claims that the Hellinger distance metric is suitable for other use cases.

Our study was performed by averaging the broad and narrow utility across 20 synthetic data sets (iterations). A larger number of iterations was evaluated (50 and 100), and we noted that the differences were not material. We opted to present the smaller number of iterations as these still give us meaningful results and would be faster computationally for others applying these results.

## Appendix

Multimedia Appendix 1 Detailed SDG method descriptions, dataset descriptions, and detailed analysis results. SDG: synthetic data generation.

## Abbreviations

AUPRC: area under the precision-recall curve
AUROC: area under the receiver operating characteristic curve
GAN: Generative Adversarial Network
LR: logistic regression
pMSE: propensity mean squared error
SDG: synthetic data generation
