# Supplementary material for: Utility Metrics for Evaluating Synthetic Health Data Generation Methods: Validation Study
Source: JMIR Med Inform. 2022 Apr 7;10(4):e35734. doi: 10.2196/35734 (PMC9030990; doi:10.2196/35734)
Supplement: Multimedia Appendix 1 [file medinform_v10i4e35734_app1.pdf]

Appendix S1: Simulation Datasets

The following datasets were used to compare the results of logistic regression between real and synthetic datasets. All of the datasets are from the UCI machine learning repository except where indicated.

Data Name	Description	Number of Records	Number of Variables	Entropy (nats)
Adult	The census income data from 1994 Census database	44842	13	1.193
BankNote	Data of images that were taken for the evaluation of tan authentication procedure for banknotes.	1371	5	1.746
Breast Cancer Wisconsin	Diagnostic Wisconsin Breast Cancer Database	683	9	1.47
Breast Cancer Coimbra	Diagnostic Coimbra Breast Cancer Database	116	9	1.558
Breast Tissue	Dataset with electrical impedance measurements of freshly excised tissue samples from the breast.	106	10	1.492
Breast Cancer	This data is provided by the Oncology Institute to predict the breast cancer.	227	10	0.975
Chronic Kidney Disease	This dataset is collected in Apollo Hospital, India. It can be used to predict the chronic kidney disease.	209	21	0.93
Heart Disease	The Cleveland heart database	303	13	1.158
Colposcopy/green	The three modalities of data are dedicated to determining two classes of the colposcopic sequences (bad, good).	98	56	1.669
Colposcopy/hinselmann		97	56	1.684
Colposcopy/schiller		92	56	1.806
Cardiotocography (3 class)	2126 fetal cardiotocograms (CTGs) were automatically processed and the respective diagnostic features measured. The CTGs were also classified by three expert obstetricians and a consensus classification label assigned to each of them. Classification was both with respect to a morphologic pattern (A, B, C. ...) and to a fetal state (N, S, P). Therefore, the dataset can be used either for 10-class or 3-class experiments.	2126	21	1.291
Cardiotocography (10 class)		2126	21	1.352
Dermatology	Aim for this dataset is to determine the type of Eryhemato-Squamous Disease.	358	34	0.89
Diabetic Mellitus	The data is dedicated to determining the type of diabetic mellitus.<br>This dataset is from OpenML.	281	97	0.56
Diabetic Retinopathy	This dataset contains features extracted from the Messidor image set to predict whether an image contains signs of diabetic retinopathy or not.	1151	19	1.044
Echocardiogram	Data for classifying if patients will survive for at least one year after a heart attack	106	10	1.534
EEGb Eye State	All data is from one continuous EEG measurement with the Emotiv EEG Neuroheadset. The data set consists of 14 EEG values and a value indicating the eye state.	14980	14	0.076
Lymphography	This lymphography domain was obtained from the University Medical Centre, Institute of Oncology,	148	18	0.818

	Ljubljana, Yugoslavia. This data is dedicated to determining the degree of lymph cancer.			
Mice Protein	This data contains 77 proteins measured in the cerebral cortex to predict 8 classes of control and Down syndrome when mice exposed to context fear conditioning.	1080	81	1.611
Postoperative Patient Data	The classification task of this database is to determine where patients in a postoperative recovery area should be sent to next.	87	8	0.741
Primary Tumor	This data is provided by the Oncology Institute to predict the tumor class (lung, head & neck, etc).	336	15	0.619
Stroke	Health care database to predict stroke	29072	10	0.887
Thoracic Surgery	The data is dedicated to the classification problem related to the post-operative life expectancy in lung cancer patients.	470	16	0.57
Thyroid Disease	The data is from the 6 databases from the Garavan Institute in Sydney, Australia. Thyroid Disease refers to Thyroid0387.data and Thyroid Dicease (new) refers to the new-thyroid.data in the UCI repository.	5786	23	0.489
Thyroid Disease (new)		215	6	1.177
Titanic_train	The train dataset on Kaggle is a subset of the passenger information on Titanic. This dataset is used to predict whether the passenger survived or not.<br>This dataset is from Kaggle.	891	8	0.778
Z-Alizadeh Sani	This data is used to predict two possible categories of CAD (normal or not normal).	303	56	0.89
Colon Cancer	The dataset was from an oncology trial, N0147, from Project Data Sphere (PDS)[1] [1].	1543	10	0.769
Colon Cancer Registry	A prospectively maintained Danish Colorectal Cancer Group (DCCG) database including all Danish patients with a first-time diagnosis of right-sided colonic cancer between 2001 and 2018 [2].[2]	12855	192	0.356

1 See <https://data.projectdatasphere.org/>
2 This dataset was obtained directly from the Danish registry and can be requested from DCCG.

Appendix S2: Additional Details on Utility Metrics

Multivariate Hellinger Distance

The Bhattacharyya distance is the degree of dissimilarity between two probability distributions [3]. It is widely used in the areas of, for example, image segmentation, and feature extraction [4],[5].

Consider the probability distributions p and q over the same domain X , the Bhattacharyya distance is defined as:

$$D_B\left(p,q\right)=-\ln\left(BC\left(p,q\right)\right) \tag{1}$$

where, the Bhattacharyya coefficient can be computed for discrete distributions as follows:

$$BC\left(p,q\right)=\sum_{x\in X}\sqrt{p\left(x\right)q\left(x\right)} \tag{2}$$

And for continuous probability distributions:

$$BC\left(p,q\right)=\int\sqrt{p\left(x\right)q\left(x\right)}dx \tag{3}$$

It is not limited to computing the similarity between two univariate distributions. For a multivariate normal distribution, the Bhattacharyya Distance is as follows:

$$D_B\left(X,Y\right)=\frac{1}{2}\log\left(\frac{\left|\Sigma_X\right|}{\sqrt{\left|\Sigma_X\right|\left|\Sigma_Y\right|}}\right)+\frac{1}{8}\left(\mu_X-\mu_Y\right)\Sigma^{-1}\left(\mu_X-\mu_Y\right)^T \tag{4}$$

where $X\sim N\left(\mu_X,\Sigma_X\right)$, $Y\sim N\left(\mu_Y,\Sigma_Y\right)$, and $\Sigma=\frac{1}{2}\left(\Sigma_X+\Sigma_Y\right)$.

The Hellinger distance can be derived from Bhattacharyya distance and has the advantage that it is bounded between zero and one, and hence is more interpretable [6]. We therefore use the Hellinger distance computed on the multivariate normal distribution.

Wasserstein Distance

In the context of optimal transport planning, the Wasserstein distance evaluates the effort it needs to transport the distribution of mass $\mu(x)$ on a space X to the distribution $v(x)$ on the same space. Given the transport plan to move from point x to point y is $\gamma(x,y)$ and the cost function is $c(x,y)$, the optimal cost can be defined as:

$$C = inf_{\gamma\in\Gamma(\mu,v)}\int c(x,y)d\gamma(x,y)$$

where, $\Gamma(\mu,v)$ denotes the collection of all measures of transports.

The optimal cost is the same as the definition of the W_1 distance if the cost function is equivalent to the distance between point x and y.

The pth Wasserstein distance between two probability distributions μ and v in $P_p(M)$ is defined as:

$$W_p(\mu, v) := \left(inf_{\gamma \in \Gamma(\mu,v)} \int_{M \times M} d(x,y)^p d\,\gamma(x,y) \right)^{\frac{1}{p}},$$

where, (M, d) is a metric space, $P_p(M)$ denotes the collection of all probability measures μ on M and $\Gamma(\mu, v)$ denotes the collection of all measures on $M \times M$ with marginals μ and v of x and y respectively.

In the current paper the W_1 metric is used.

Distinguishability

After stacking the real and synthetic datasets, using a binary classifier has been proposed as an approach for comparing two multivariate distributions [7], [8]. The estimated probability across all observations would then be used to compute a score. In our context the two datasets would be the real and synthetic datasets.

Adopting a perspective from the propensity score matching literature [9], a propensity mean square error metric has been proposed to evaluate the similarity of real and synthetic datasets [10], [11], which we will refer to as *propensityMSE*. To calculate the *propensityMSE*, a classifier is trained on a stacked dataset consisting of real observations labelled 1 and synthetic observations labelled 0. The *propensityMSE* score is computed as the mean squared difference of the estimated probability from the average prediction where it is not possible to distinguish between the two datasets. If the datasets are of the same size and indistinguishable, which is the assumption we make here, the average estimate will be 0.5:

$$propensityMSE = \frac{1}{N}\sum_{i}(p_i - 0.5)^2 \tag{5}$$

where N is the size of the stacked dataset, and p_i is the propensity score for observation i. The classifier is used to compute the p_i value where the training set is also used to compute the propensity score for each observation in the stacked dataset.

If the multivariate distributions of the two datasets are the same, then the probability will hover around 0.5, indicating that the classifier is not able to distinguish between them and the *propensityMSE* approaches zero. If the two datasets are completely different, then the classifier will be able to distinguish between them. In such a case the propensity score will be either zero or one, with *propensityMSE* approaching 0.25.

To make the metric more easily interpretable we can scale it to be between zero and one as follows (for the case when the two datasets are the same size):

$$\frac{1}{N}\sum_{i}\frac{(p_i - 0.5)^2}{0.25} \tag{6}$$

Another related approach that has been used to evaluate the utility of synthetic data is to take a prediction perspective rather than a propensity perspective. This has been applied with “human

discriminators" by asking a domain expert to manually classify sample records as real or synthetic [12]–[14]. This means that a sample of real records and a sample of synthetic records are drawn, and the two sets are shuffled together. Then the shuffled records are presented to clinicians who are expert in the domain and asked to subjectively discriminate between them by indicating which record is real versus synthetic. In this task the classification of every record is either correct or incorrect. A correct classification is not good in this case because it indicates that the real and synthetic data are more distinguishable. A real record that is classified as synthetic and a synthetic record that is classified as real are both considered good outcomes because the clinician was not able to tell the difference. High distinguishability only occurs when the human discriminator can correctly classify real and synthetic records.

The use of human discriminators is not scalable and therefore we can use machine learning algorithms trained on a training dataset and that make predictions on a hold-out test dataset. This approach mimics the subjective evaluations described above. We will refer to this metric as *predictionMSE*. Also note that this is different than the calculation of *propensityMSE* where the training dataset is also used to compute the probabilities.

The *predictionMSE* calculation needs to be adjusted so as not to penalize incorrect classification. For example, if a real record has a predicted probability less than 0.5 then this would be penalized under *propensityMSE*, but under the prediction approach this is an indicator that the discriminator is unable to distinguish between real and synthetic records. We therefore define the *predictionMSE*:

$$g_i = \begin{cases} p_i^{real}: & \left(\max\left(p_i^{real}, 0.5\right) - 0.5\right)^2 \Big/ 0.25 \\ p_i^{syn}: & \left(\min\left(p_i^{syn}, 0.5\right) - 0.5\right)^2 \Big/ 0.25 \end{cases} \quad (7)$$

where the full adjusted metric would be:

$$predictionMSE = \frac{1}{N}\sum_{i=1}^{N} g_i \quad (8)$$

This formulation does not penalize synthetic data that looks more like real data than it does like synthetic data. The concept of using prediction error for the stacked dataset has been considered before, but AUROC was used rather than the squared error [15].

Appendix S3: Analysis Results

Sequential Synthesis

Data	hellinger	lgc[3]	mmd[4]	propenMSE	predMSE	wass[5]	auroc	auprc
**1**	0.053520735	-9.411165012	0.000445095	0.063772899	0.053446777	165.0590447	0.014289951	0.012558837
**2**	0.077104139	-8.450672221	0.00042547	0.001539146	0.000498051	0.300491483	0.050477409	0.023283874
**3**	0.12815548	-6.505201868	0.000178482	0.00018475	0.000102075	0.330844802	0.013706406	0.007774163
**4**	0.467965021	-5.650757509	0.01721333	0.001801156	0.00115298	6.433722005	0.058819665	0.047396561
**5**	0.896110992	-4.690169409	0.019045602	0.002401078	0.001153406	213.6377703	0.03686043	0.058839617
**6**	0.189015506	-6.88790092	0.000395217	0.00044786	0.000208898	0.093797834	0.039262886	0.077835779
**7**	0.680869209	-5.560410447	0.0095941	0.168007941	0.030325163	1.359139495	0.045159706	0.159459477
**8**	0.25943993	-6.308478662	0.006487143	0.001759728	0.000791107	1.381272277	0.021854095	0.031310656
**9**	1	-4.288180419	0.0204082	0.617698875	0.231393248	2.890557495	0.048395691	0.058707205
**10**	1	-4.776083365	0.020618557	0.6373706	0.221576216	2.467043432	0.073541071	0.046952292
**11**	0.999999998	-5.321196126	0.02173913	0.461406874	0.156158077	2.757909118	0.054966854	0.060608759
**12**	0.842158376	-6.051642953	0.001028988	0.962573962	0.768830604	2.014853545	0.135038934	0.23998322
**13**	0.845818418	-6.040428371	0.001028104	0.967678137	0.769551358	2.05258473	0.099651539	0.281608028
**14**	0.604983569	-4.932445678	0.006167889	0.008734815	0.00257874	1.067555467	0.051260013	0.148542056
**15**	0.997946564	-6.664806914	NA	0.035667583	0.006907523	0.964773114	0.035269574	0.030341733
**16**	0.966570909	-5.233848218	0.001836148	0.96209672	0.882134843	3.419219013	0.134013655	0.044748912
**17**	0.562561483	-4.975351575	NA	0.002751035	0.001503369	0.900174099	0.041571351	0.051815112
**18**	0.248562702	-2.564739323	0.001335127	0.68967367	0.656936531	11.8777124	0.06192864	0.060721724
**19**	0.525627846	-6.047856774	0.001409056	0.000668806	0.000337814	0.079576814	0.103612114	0.152366732
**20**	1	-6.395720729	NA	0.999878441	0.951256071	0.022862158	0.000227743	0.381523647
**21**	0.315461114	-1.451784042	0.021027174	0.000597488	0.000262358	1.168186462	0.099614175	0.154500325

[3] We took the log of the U_c value to be consistent with the original article [16].

[4] Because the variables need to be converted to a binary representation, some of the less frequent categories are not generated in the synthetic datasets, which causes the calculation to be invalid.

[5] For nominal variables, the integer encoding can result in inflated distances.

**22**	0.300167914	-4.962788354	0.001248801	0.000616819	0.000304652	0.081233259	0.023009008	0.030891455
**23**	0.025831233	-10.88640466	0.000691433	0.006021694	0.003096619	0.394039559	3.51E-07	0.005865767
**24**	0.272949363	-5.591368389	0.006227206	0.981999755	0.973084005	0.154295207	0.046806065	0.089173389
**25**	0.382372323	-7.540376921	0.000358852	0.99901793	0.998757018	0.274196256	0.024998977	0.02626433
**26**	0.16795082	-5.858555927	0.009809507	0.009599928	0.001625138	0.698603101	0.047616289	0.126904782
**27**	0.07271509	-6.92736412	1.11E-03	0.311748242	0.171260301	0.772405438	0.013403708	0.02503817
**28**	0.911068385	-6.078973345	NA	0.543422229	0.058967994	6.158849287	8.76E-02	0.110049977
**29**	0.153798176	-7.10573017	0.015839619	0.015707364	0.009671787	0.219053806	0.006509183	0.028674618
**30**	0.042289564	-7.16299483	3.71E-04	0.004852046	0.002616708	0.139621066	0.00281873	0.010876145

CTGAN

Data	hellinger	lgc	mmd	propenMSE	predMSE	wass	auroc	auprc
1	0.114483375	-4.92460737	0.000412667	0.942911862	0.925443096	1483.063644	0.007460151	0.020115969
2	0.50364019	-3.346062334	0.035635267	0.922584196	0.803053734	0.844088564	0.292108274	0.154058627
3	0.589353543	-2.165395963	0.082285033	0.80708701	0.710988142	0.46042899	0.31755332	0.251222487
4	0.706602114	-2.89211302	0.017240961	0.878330067	0.642856294	38.03062978	0.189449167	0.078931258
5	0.973071501	-2.527951679	0.019045924	0.985246851	0.858046792	563.2459919	0.361184207	0.323248359
6	0.343194815	-5.446315908	0.00172322	0.27547596	0.112448552	0.140075812	0.116240159	0.08230844
7	0.744079066	-3.557856353	0.009593388	0.964519492	0.803766204	3.864892562	0.408745126	0.553150876
8	0.475731179	-4.293722943	0.00663916	0.657444536	0.481239152	3.287835455	0.308963128	0.400604508
9	1	-2.119851881	0.0204082	0.999826403	0.922598855	9.727659953	0.049230841	0.271274527
10	1	-1.585258751	0.020618557	0.999940445	0.962376972	11.76671547	0.061378012	0.213130033
11	1	-2.136444061	0.02173913	0.999935813	0.960805353	9.753606485	0.055330262	0.297747608
12	0.945607165	-4.159615247	0.001021587	0.998949283	0.965145208	3.861047977	0.03948376	0.12840464
13	0.922793004	-4.812829603	0.001020807	0.999305794	0.968650885	4.458988797	0.202588274	0.396001754
14	0.855410008	-2.690568045	0.013108804	0.930628412	0.687955237	0.621264166	0.48746811	0.703488009
15	0.99930741	-3.620974025	0.007189938	0.997310194	0.838966978	0.290532682	0.285438568	0.085361896
16	0.997453158	-2.392738625	0.001836262	0.999904229	0.986829569	5.509245232	0.158306267	0.08664414
17	0.641508173	-2.721228933	0.021166222	0.873988073	0.657072645	2.56066187	0.244748886	0.191869817
18	0.61638154	-1.896836603	0.001335127	0.999663989	0.985879664	19.34272946	0.035906247	0.171503645
19	0.632699015	-5.561445351	0.003486727	0.360246968	0.19090567	0.099121622	0.284860139	0.446442718
20	1	-4.42765826	0.021292023	0.999992718	0.99689002	0.078135807	0.394712901	0.766599768
21	0.326164979	-5.156927554	0.001416157	0.009776574	0.009712449	0.108812261	0.06715464	0.130947453
22	0.35569447	-6.637024311	0.001792854	0.432367501	0.255786179	0.072700893	0.271238247	0.193921703
23	0.106820164	-4.90254152	0.00096344	0.235050664	0.189466601	1.328482445	0.003472501	0.162385569
24	0.469061301	-2.912560181	NA	0.999335541	0.998298167	0.594054005	0.020486876	0.118627977
25	0.718930696	-3.87232962	NA	0.999999197	0.999938352	1.136337672	0.319773476	0.366386107
26	0.448587653	-2.777350063	0.040231065	0.923121131	0.788543478	3.904886047	0.414553415	0.500295993
27	0.346112085	-4.910507285	0.019317847	0.730263256	0.600534187	1.23782129	0.257562394	0.346412341

**28**	0.976392787	-4.908078906	0.00660066	9.97E-01	0.823461188	12.20846239	0.226371585	0.207017498
**29**	0.144411441	-4.286725491	0.04286848	0.293045441	0.238957138	0.913382533	0.009621811	0.210900816
**30**	0.158231201	-5.477202961	0.001250063	0.255206719	2.03E-01	0.390392876	0.071639984	0.236903452

Bayesian Network

Data	hellinger	lgc	mmd	propenMSE	predMSE	wass	auroc	auprc
1	0.247133562	-1.9064696	0.000412705	0.99575166	0.989854649	37744.47505	0.082919889	0.09295752
2	0.546767984	-2.3569115	NA	0.922517815	0.842497793	2.135548451	0.488380603	0.48823881
3	0.620147895	-2.1709452	NA	0.835345025	0.735075952	2.11704978	0.452903126	0.484087882
4	0.706877704	-3.0243963	NA	0.914661437	0.735426612	44.70093089	0.191240392	0.299517404
5	0.973602548	-1.733155	0.019045924	0.869269379	0.782275363	8143.479319	0.380047925	0.370618267
6	0.348764067	-3.1971974	0.021386195	0.770651214	0.619492458	0.476064982	0.079478214	0.092450051
7	0.75765177	-2.1731502	0.009598477	0.969778069	0.828412116	12.07446972	0.371605613	0.333196972
8	0.469325336	-2.2965761	NA	0.908070978	0.736686325	10.67467704	0.310263611	0.364599119
9	1	-1.8776601	NA	0.989955162	0.94360149	9.562090028	0.048398799	0.188307684
10	1	-1.7812539	NA	0.98628881	0.922617054	12.23948368	0.062601058	0.324900397
11	1	-2.4406461	NA	0.998291372	0.921463594	8.195475787	0.046430035	0.229417971
12	0.967467176	-1.5581497	NA	0.999735183	0.994236139	26.8873786	0.445550453	0.503271638
13	0.968117521	-1.5541744	NA	0.99918262	0.993854011	26.9300682	0.457859832	0.606196385
14	0.861416337	-3.5935751	NA	0.994868566	0.956769321	0.98782921	0.309035257	0.577678412
15	0.999770708	-4.3859152	NA	0.998552584	0.977524892	0.486262223	0.120542492	0.383953935
16	0.997529541	-1.9289823	NA	0.997561483	0.987042013	21.19668054	0.247726476	0.327538791
17	0.651043863	-3.2716399	0.020593438	0.764573289	0.509284513	2.251553063	0.266233958	0.210578485
18	0.921614221	-1.581402	NA	0.999539015	0.999006253	23010.45159	0.126891127	0.153364621
19	0.636603249	-2.9692647	NA	0.834990844	0.648599859	0.452098151	0.29894258	0.540588039
20	1	-1.9220571	0.172137511	0.999631314	0.995420314	0.23276242	0.50199115	0.847716673
21	0.357696977	-2.664996	0.046520097	0.654625026	0.546801785	0.336845466	0.117524729	0.140452779
22	0.37542924	-4.2188914	NA	0.892057837	0.787368235	0.44360119	0.233348002	0.215023441
23	0.259998707	-3.1638624	0.001044381	0.601975991	0.551779301	4.554311152	0.127687949	0.467034189
24	0.483444959	-1.7449915	0.093779355	0.979699735	0.947815631	3.413553867	0.04160945	0.317904229
25	0.77978103	-1.506563	0.000582576	0.997410932	0.988127555	25.10613017	0.317784229	0.421103049
26	0.454107775	-1.860538	NA	0.968936451	0.912496533	11.15575216	0.482978196	0.547865664
27	0.332591925	-1.8105487	0.138999649	0.939601052	0.892255256	36.16548851	0.206866136	0.225963344

**28**	0.97414672	-2.8528429	0.00660066	0.999390804	0.984422712	74.69310922	0.199617732	0.052584344
**29**	0.187659027	-2.9485405	3.74E-02	0.763302205	0.677658511	2.079594999	0.130740637	0.392491527
**30**	0.350916214	-1.5083984	0.009491316	0.963919095	0.950507062	43.53385604	0.403687728	0.684383417

Appendix S4: Prediction Accuracy

The following synthetic data values are the average across 20 synthetic datasets. They were used to compute the AUROC and AUPRC differences. Note that for each SDG method the real data LR model was re-estimated and therefore the values are slightly different due to cross-validation partitions being different.

Sequential Synthesis

Data	real_auroc	syn_auroc	real_auprc	syn_auprc
1	0.758337414	0.743908559	0.747931	0.734633
2	0.989529692	0.938038253	0.999699	0.977657
3	0.962829392	0.950075618	0.989	0.983208
4	0.726677489	0.699180167	0.811209	0.809474
5	0.875631488	0.85527904	0.536727	0.524573
6	0.624840866	0.660369968	0.464413	0.57346
7	0.916044554	0.876634379	0.934816	0.764252
8	0.826387181	0.814547615	0.875132	0.898516
9	0.564650856	0.561451798	0.710358	0.671833
10	0.536479277	0.563003629	0.845624	0.82235
11	0.554463109	0.57319603	0.745436	0.722516
12	0.948697211	0.81044978	0.840116	0.596171
13	0.961375359	0.861880061	0.713498	0.429498
14	0.988357847	0.934316063	0.882512	0.724672
15	0.792289994	0.792534261	0.891474	0.885999
16	0.750482536	0.610760269	0.86869	0.820992
17	0.773001323	0.791179201	0.539803	0.555758
18	0.627072241	0.563712368	0.619397	0.553989
19	0.78443563	0.704173678	0.790395	0.662167
20	0.999931141	0.999869425	0.976136	0.593892
21	0.473868874	0.555334478	0.25829	0.381852
22	0.774262461	0.76288002	0.288286	0.279971
23	0.5	0.5	0.082271	0.077881
24	0.514483101	0.553213083	0.238643	0.297639
25	0.966776054	0.941499854	0.496138	0.489429

**26**	0.993204419	0.947282481	0.897648	0.773224
**27**	0.772477158	0.76950541	0.798916	0.800872
**28**	0.727708789	0.670285682	0.524958	0.454522
**29**	0.509587395	0.50387905	0.254459	0.231487
**30**	0.507783829	0.50832476	0.219282	0.216995

CTGAN

Data	real_auroc	syn_auroc	real_auprc	syn_auprc
**1**	0.758498	0.765654	0.748106	0.767564
**2**	0.988996	0.698383	0.999679	0.848532
**3**	0.961307	0.63796	0.98916	0.736829
**4**	0.728841	0.52352	0.814177	0.749789
**5**	0.880221	0.509748	0.54155	0.203446
**6**	0.61757	0.491042	0.458533	0.374704
**7**	0.912884	0.514221	0.923662	0.371159
**8**	0.823341	0.513056	0.873446	0.473714
**9**	0.550962	0.558486	0.70113	0.420435
**10**	0.54903	0.550932	0.856719	0.625126
**11**	0.577954	0.562201	0.749689	0.448526
**12**	0.948226	0.988671	0.840835	0.970665
**13**	0.96142	0.758892	0.712225	0.314424
**14**	0.989736	0.494586	0.887725	0.18128
**15**	0.792394	0.500926	0.885807	0.796553
**16**	0.749719	0.592164	0.868401	0.779497
**17**	0.773104	0.538805	0.536377	0.356569
**18**	0.626466	0.663247	0.619184	0.791573
**19**	0.787497	0.506575	0.783802	0.34877
**20**	0.999968	0.600866	0.97701	0.205062
**21**	0.482896	0.524985	0.249028	0.386454
**22**	0.770301	0.497383	0.284825	0.095026
**23**	0.5	0.503191	0.082591	0.244358

**24**	0.518816	0.501898	0.238685	0.340603
**25**	0.967171	0.640571	0.493994	0.126236
**26**	0.993856	0.578109	0.904026	0.404304
**27**	0.773741	0.510273	0.803527	0.44426
**28**	0.737133	0.50174	0.535839	0.328978
**29**	0.5095	0.5	0.252718	0.034132
**30**	0.507623	0.580011	0.219697	0.457873

Bayesian Network

Data	real_auroc	syn_auroc	real_auprc	syn_auprc
**1**	0.758142	0.675311	0.747922	0.65496
**2**	0.989277	0.507335	0.999694	0.512796
**3**	0.959741	0.510917	0.988631	0.510332
**4**	0.725227	0.543033	0.811434	0.530498
**5**	0.875808	0.479279	0.535723	0.169572
**6**	0.608826	0.527565	0.454086	0.547506
**7**	0.917295	0.536314	0.938928	0.597919
**8**	0.816592	0.520897	0.862298	0.500824
**9**	0.581897	0.552274	0.71187	0.497205
**10**	0.523543	0.547317	0.852866	0.524696
**11**	0.557279	0.55112	0.731765	0.522933
**12**	0.948642	0.500249	0.841233	0.336071
**13**	0.960547	0.500656	0.710127	0.102175
**14**	0.988147	0.679997	0.886689	0.304223
**15**	0.791333	0.647315	0.88975	0.493995
**16**	0.748336	0.496167	0.867405	0.537961
**17**	0.774246	0.512317	0.543819	0.342775
**18**	0.627062	0.499847	0.619283	0.465439
**19**	0.788076	0.494728	0.796475	0.254498
**20**	0.999832	0.491398	0.975738	0.125457
**21**	0.461107	0.582628	0.255496	0.397078

**22**	0.772113	0.529853	0.284631	0.068543
**23**	0.5	0.627472	0.082408	0.549978
**24**	0.514713	0.548012	0.23995	0.558268
**25**	0.96762	0.648961	0.495363	0.073739
**26**	0.99432	0.510752	0.903662	0.34683
**27**	0.772119	0.568908	0.799393	0.571657
**28**	0.735188	0.529235	0.531059	0.567726
**29**	0.50931	0.635766	0.253335	0.643381
**30**	0.508237	0.911422	0.219738	0.903405

Appendix S5: Results Plots

The following are the plots showing the prediction performance rank for the utility metrics not shown in the main body of the paper. For all the plots the three SDG methods were ordered based on their relative utility metric values into the "H", "M", and "L" groups.

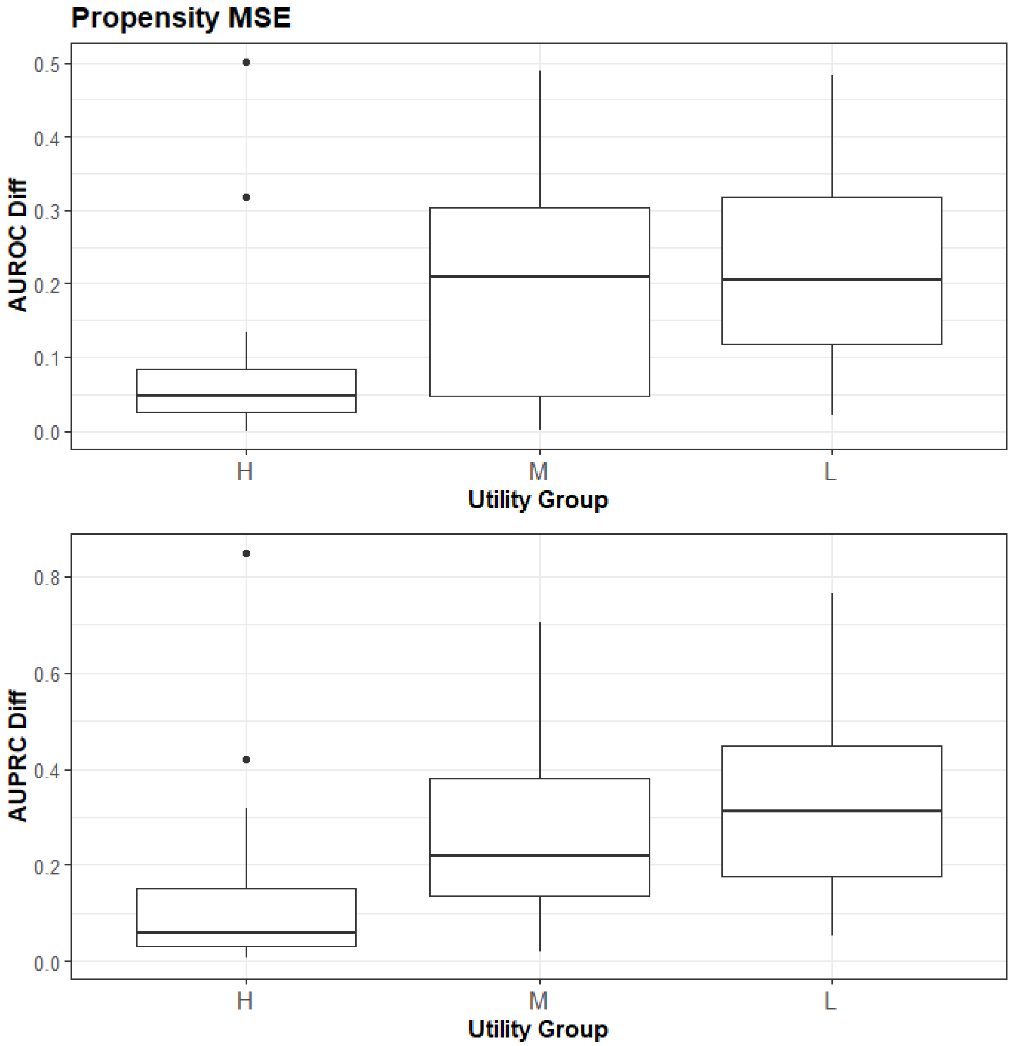

Figure 1: The relationship between the propensity MSE vs the AUROC and AUPRC.

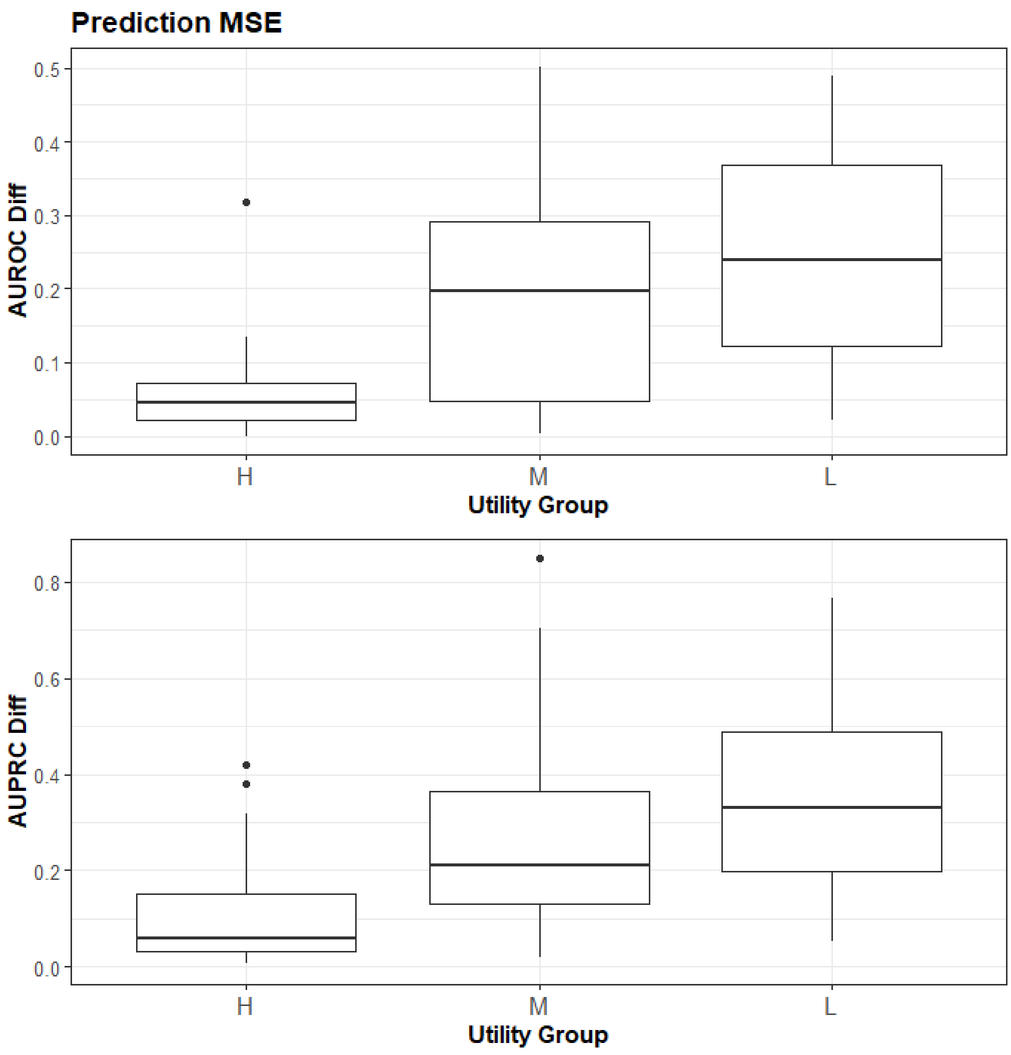

Figure 2: The relationship between the prediction MSE vs the AUROC and AUPRC.

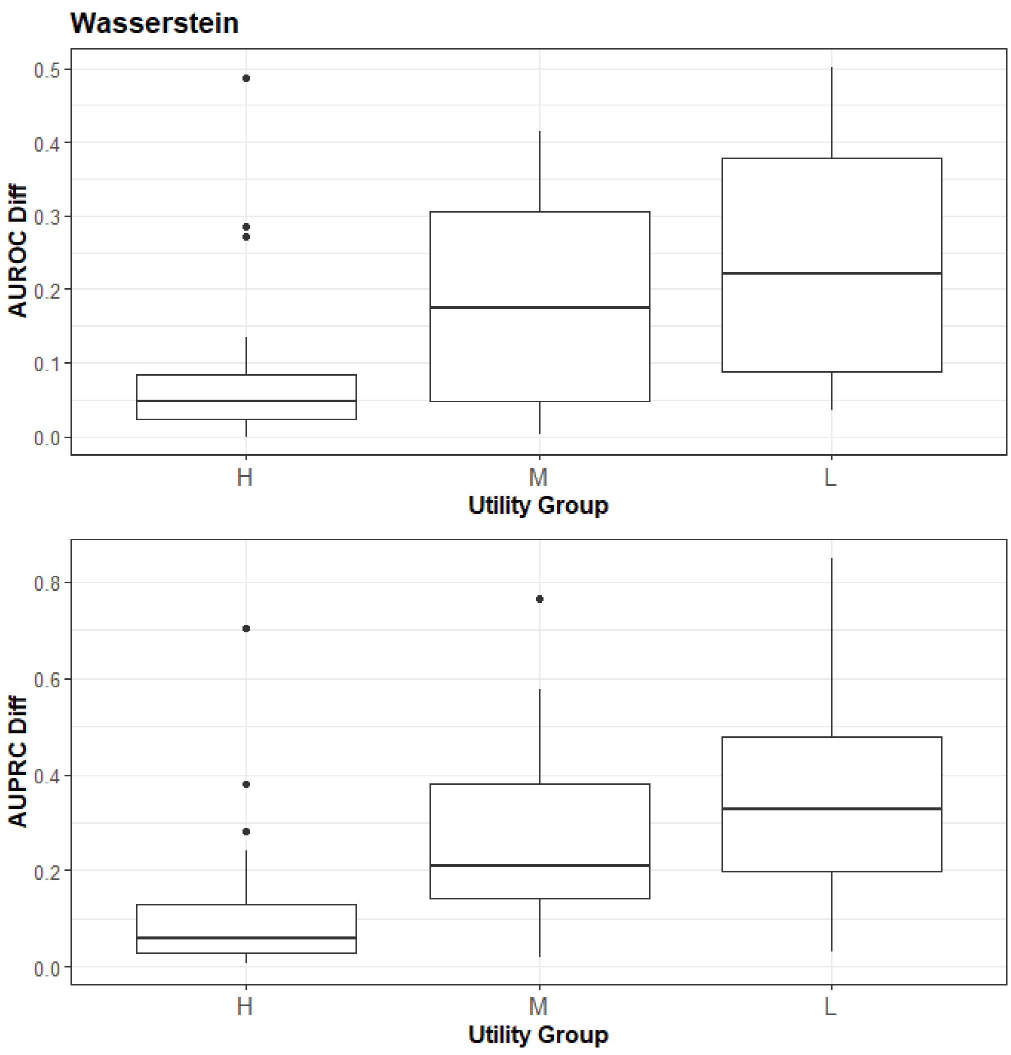

Figure 3: The relationship between the Wasserstein distance vs the AUROC and AUPRC.

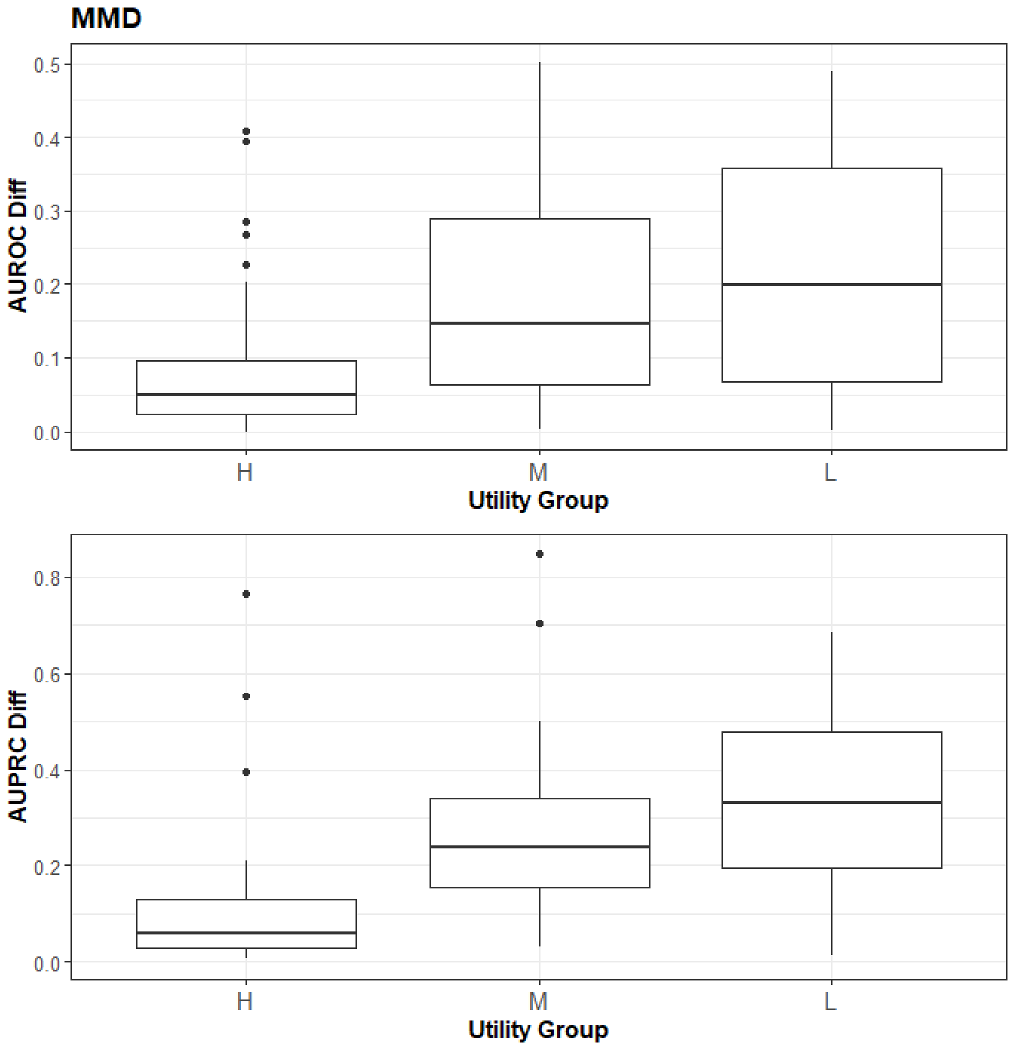

Figure 4: The relationship between MMD vs the AUROC and AUPRC.

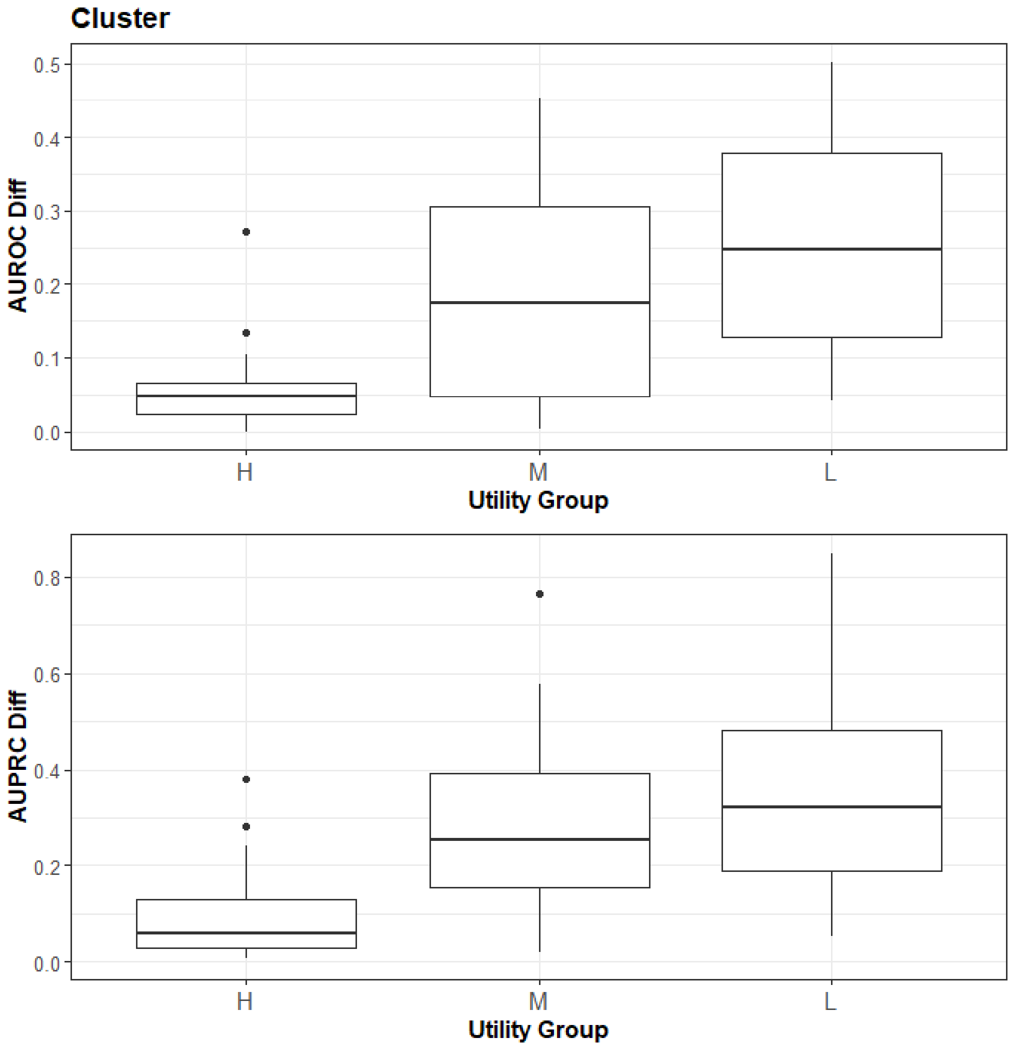

Figure 5: The relationship between the cluster distance vs the AUROC and AUPRC.

References

[1] CEO Life Sciences Consortium, “Share, Integrate & Analyze Cancer Research Data | Project Data Sphere.” https://projectdatasphere.org/projectdatasphere/html/home

[2] A. El-Hussuna, T. Lytras, N. H. Bruun, M. F. Klein, S. H. Emile, and N. Qvist, “Extended Right-Sided Colon Resection Does Not Reduce the Risk of Colon Cancer Local-Regional Recurrence: Nationwide Population-Based Study from Danish Colorectal Cancer Group Database,” *Diseases of the Colon & Rectum*, p. 10.1097/DCR.0000000000002358, 2022, doi: 10.1097/DCR.0000000000002358.

[3] Anil Kumar Bhattacharya, “On a Measure of Divergence between Two Multinomial Populations,” *The Indian Journal of Statistics*, vol. 7, no. 4, pp. 401–406, 1946.

[4] Oleg Michailovich, Yogesh Rathi, and Allen Tannenbaum, “Image Segmentation Using Active Contours Driven by the Bhattacharyya Gradient Flow,” *IEEE Transactions on Image Processing*, vol. 16, no. 11, pp. 2787–2801, 2007, doi: 10.1109/TIP.2007.908073.

[5] Euisun Choi and Chulhee Lee, “Feature extraction based on the Bhattacharyya distance,” *Pattern Recognition*, vol. 36, no. 8, pp. 1703–1709, 2003, doi: https://doi.org/10.1016/S0031-3203(03)00035-9.

[6] K. G. Derpanis, “The Bhattacharyya Measure.” 2008.

[7] Jerome Friedman, “On Multivariate Goodness-of-Fit and Two-Sample Testing,” Stanford University, 2003.

[8] S. Hediger, L. Michel, and J. Näf, “On the Use of Random Forest for Two-Sample Testing,” *arXiv:1903.06287 [stat]*, Apr. 2019, Accessed: May 06, 2020. [Online]. Available: http://arxiv.org/abs/1903.06287

[9] P. R. Rosenbaum and D. B. Rubin, “The central role of the propensity score in observational studies for causal effects,” *Biometrika*, vol. 70, no. 1, pp. 41–55, Apr. 1983, doi: 10.1093/biomet/70.1.41.

[10] M.-J. Woo, J. P. Reiter, A. Oganian, and A. F. Karr, “Global Measures of Data Utility for Microdata Masked for Disclosure Limitation,” *Journal of Privacy and Confidentiality*, vol. 1, no. 1, Apr. 2009, doi: 10.29012/jpc.v1i1.568.

[11] J. Snoke, G. M. Raab, B. Nowok, C. Dibben, and A. Slavkovic, “General and specific utility measures for synthetic data,” *Journal of the Royal Statistical Society: Series A (Statistics in Society)*, vol. 181, no. 3, pp. 663–688, Jun. 2018, doi: 10.1111/rssa.12358.

[12] B. K. Beaulieu-Jones, Z. S. Wu, C. Williams, and C. S. Greene, “Privacy-preserving generative deep neural networks support clinical data sharing,” *bioRxiv*, p. 159756, Jul. 2017, doi: 10.1101/159756.

[13] E. Choi, S. Biswal, B. Malin, J. Duke, W. F. Stewart, and J. Sun, “Generating Multi-label Discrete Patient Records using Generative Adversarial Networks,” in *Machine Learning for Healthcare Conference*, 2017, vol. 68, pp. 286–305. [Online]. Available: http://proceedings.mlr.press/v68/choi17a/choi17a.pdf

[14] A. Salim Jr, “Synthetic Patient Generation: A Deep Learning Approach Using Variational Autoencoders,” *arXiv:1808.06444 [cs, stat]*, Aug. 2018, Accessed: Aug. 06, 2021. [Online]. Available: http://arxiv.org/abs/1808.06444

[15] G. Muniz-Terrera, O. Mendelevitch, R. Barnes, and M. D. Lesh, “Virtual Cohorts and Synthetic Data in Dementia: An Illustration of Their Potential to Advance Research,” *Front. Artif. Intell.*, vol. 4, 2021, doi: 10.3389/frai.2021.613956.

[16] A. Goncalves, P. Ray, B. Soper, J. Stevens, L. Coyle, and A. P. Sales, “Generation and evaluation of synthetic patient data,” *BMC Med Res Methodol*, vol. 20, no. 1, p. 108, Dec. 2020, doi: 10.1186/s12874-020-00977-1.
